# HPV impacts survival of stage IVC non-oropharyngeal HNSCC cancer patients

**DOI:** 10.15761/OHNS.1000160

**Published:** 2018-02-24

**Authors:** Adam R Burr, Paul M Harari, Huaising C Ko, Shuai Chen, Menggang Yu, Andrew M Baschnagel, Randall J Kimple, Matthew E Witek

**Affiliations:** 1 Department of Human Oncology, School of Medicine and Public Health, University of Wisconsin, Carbone Cancer Center, Madison, WI, USA; 2 Department of Biostatistics and Medical Informatics, School of Medicine and Public Health, University of Wisconsin, Carbone Cancer Center, Madison, WI, USA

**Keywords:** HPV, head and neck cancer, oropharynx

## Abstract

**Objectives:**

Human papillomavirus (HPV) status is a favorable prognostic marker for patients with oropharyngeal squamous cell carcinoma (OPSCC) and non-metastatic head and neck non-OPSCC. We evaluated the impact of HPV status on overall survival (OS) for patients with Stage IVC non-OPSCC.

**Materials and methods:**

Patients diagnosed with Stage IVC non-OPSCC and known HPV status between 2010–2013 were identified in the National Cancer Database. Univariate and multivariate analyses were performed to determine factors associated with OS. Propensity score-weighted Kaplan-Meier estimation was used to adjust for confounders in OS analyses. Multiple imputation method was used for sensitivity analysis.

**Results:**

We identified 708 patients with Stage IVC non-OPSCC with 30% being HPV-positive. Unadjusted median survival was 10.3 months for HPV-negative patients and 21.4 months for HPV-positive patients (p<0.0001). Age ≥ 65 and tumor diameter were associated with worse OS (p<0.05) while treatment versus no treatment and HPV-positive status were associated with improved OS on multivariate analysis (p<0.001). Adjusted median survival for patients with HPV-negative and HPV-positive disease was 11.1 months and 23.8 months, respectively (p<0.001). On unadjusted subgroup analysis, patients with HPV-positive oral cavity disease exhibited improved outcomes (p<0.0001) while HPV-positive hypopharynx (p<0.06) and larynx (p<0.12) patients exhibited a trend for improved OS compared to HPV-negative patients. The survival advantage associated with HPV positivity was maintained on sensitivity analysis (p<0.01).

**Conclusion:**

These data demonstrate a clinically meaningful association between HPV status and OS in patients with non-OSPCC presenting with Stage IVC disease. In the absence of randomized data, these findings support active consideration of HPV status in clinical decision making, clinical trial design, and patient counseling regarding prognosis.

## Introduction

Human papillomavirus (HPV) is a powerful independent marker for patients with oropharyngeal squamous cell carcinoma (OPSCC). Patients with HPV-positive OPSCC exhibit a reduction in the risk of death by approximately half of that compared to patients with HPV-negative tumors [1-4]. Despite these improved outcomes, 10-30% of patients will experience disease progression within 3 years following completion of their definitive therapy [5-7]. Similar to treatment naïve patients, HPV detection remains a predictor of improved overall survival for patients who experience locoregional and/or distant disease progression following initial treatment [8,9].

Recent data supports similar favorable prognostic implications of HPV status for patients with non-OPSCC primary cancers. A combined analysis of RTOG trials 0129, 0234, and 0522 reported that p16 expression, a surrogate marker of HPV, was associated with improved overall survival and progression-free survival in patients with non-OPSCC [10]. In addition, several individual series have reported similar results ^11-13^. In a single institution series evaluating oral cavity squamous cell carcinoma, p16 was overexpressed in 44% of patients examined and shown to be a favorable prognostic factor correlating with improved overall survival and relapse-free survival [11]. In another series, patients with HPV/p16 positive hypopharyngeal or laryngeal squamous cell carcinoma undergoing definitive therapy exhibited 2-year disease free (DFS) and local recurrence free survival (LRFS) of 100% when treated with concurrent chemoradiotherapy in marked contrast to HPV/p16 negative patients with 2-year DFS and LRFS rates of 68% and 72%, respectively [12]. A more recent series supported the favorable prognostic implications of p16 over-expression in SCC from an unknown primary compared with those that were p16-negative [13].

We recently reported outcomes for patients identified in the National Cancer Database (NCDB) with early stage and locally advanced HPV-negative and HPV-positive non-OPSCC [14]. Adjusted 3-year overall survival for HPV-negative and HPV-positive non-OPSCC was 62% and 80%, respectively. These findings were consistent among oral cavity, hypopharynx, and larynx subsites. Improved outcomes were independent of clinical risk groupings although the extent of difference was more evident in patients with locally advanced compared to early stage disease. This reduction in death was similar to patients with non-metastatic HPV-positive OPSCC. Given previous data regarding the clinical implication of HPV-status in patients with metastatic OPSCC [8], we evaluated the impact of HPV status on patients presenting with Stage IVC non-OPSCC.

## Materials and methods

### Data source and patient selection

A retrospective, cohort study using data from the NCDB was performed [13]. The NCDB contains patient demographics, disease characteristics, and initial cancer treatments received from over 1,500 American College of Surgeons Commission on Cancer-accredited programs. The American College of Surgeons Commission on Cancer have not verified the data and are not responsible for the analytical and statistical methodology used and/or the conclusions reached. Overall survival is the only available outcomes data. Independent variables included patient age, sex, and truncated Charlson/Deyo comorbidity scores (CDCS; range, 0 to ≥ 2, with higher scores indicating more comorbidities). Patient demographics included income, race, facility location as urban or nonurban, year of diagnosis, and insurance status. Treatment facility information was further categorized by facility volume and community vs academic. NCDB coding instructions for HPV status require the recording of any result from an HPV tests performed on pathologic specimens from a primary tumor or metastatic site including lymph nodes. Patient HPV classifications as defined by the NCDB are detailed in Table 1.

### Statistical analysis

The primary outcome was overall survival, which was defined as the date of diagnosis to date of death. Baseline demographics and patient characteristics were analyzed by Pearson chi-square tests except for age and tumor size, which were considered continuous variables and therefore analyzed with Wilcoxon signed-rank test. Multivariate logistic regression was performed with stepwise variable selection and applied to patient and tumor characteristics to further examine factors associated with occurrence of HPV positivity.

The Kaplan–Meier method was used to compare survival outcomes between HPV-positive and HPV-negative groups. Univariate survival analysis and multivariate analysis were performed with Cox proportional hazards models using overall survival as the outcomes. Factors found to be significant in univariate analysis were included and selected by stepwise selection in multivariate analysis. The proportional hazards assumption was checked using the test based on Schoenfeld residuals. The proportionality assumption was not violated in the final selected multivariate model.

To account for confounding and covariate imbalances between HPV-positive and HPV-negative groups, we used propensity score-weighted Kaplan-Meier estimator, where the probability of HPV-positivity, the propensity score, was estimated using multivariate logistic regression. Significant covariates in multivariate survival analysis were used in the propensity score model, which included age, tumor size, and any treatment. Note that, HPV is not an actual ‘treatment’ in the conventional sense of causal inference since it is not able to be manipulated. The propensity score is used here as a tool to balance the covariate distribution between groups for studies with either causal or non-causal purposes [15]. We evaluated the distribution of propensity scores for each HPV group and confirmed sufficient overlap in the distributions. We then grouped patients into quintiles according to their estimated propensity scores and used the Cochran-Mantel-Haenszel test to verify that covariates were balanced across all strata.

The above analyses were limited to patients with known HPV status. Thus, additional sensitivity analyses were performed prior to removing patients with unknown HPV status. We used the multiple imputation method based on missing at random (MAR) assumption for patients with unknown HPV status, [16]. All analyses were performed using 3.2.2 (*R Foundation for Statistical Computing, Vienna, Austria*) SAS 9.4 (*SAS Institute Inc., Cary, NC*). All p-values were two-sided, and a p ≤ *0.05* was considered statistically significant.

## Results

### Patients

We identified 708 patients diagnosed between 2011–2013 with Stage IVC non-OPSCC of which 494 and 214 were HPV-negative and HPV-positive, respectively. Baseline patient demographics and disease characteristics are described in Table 1 with statistically significant differences noted between patient age, sex, insurance type, income, facility volume, head and neck subsite, radiotherapy dose, primary treatment, and T-stage. Statistically significant differences were noted for patients with HPV-positive disease as they were younger, male, had fewer comorbidities, carried private insurance, had higher incomes, and presented more frequently with oral cavity primaries (Table 2). Statistical significance was reached when comparing T-stage as HPV-negative patients were diagnosed with ≥ T3 tumors in 57% of cases while patients with HPV-positive disease presented with ≥ T3 tumors in 47% of cases (p<0.05). There was no statistically significant difference in nodal disease burden between HPV-negative and HPV-positive cohorts with the majority of patients presenting with ≥ N2b disease (p=0.15).

### Outcomes

**Figure 1:**
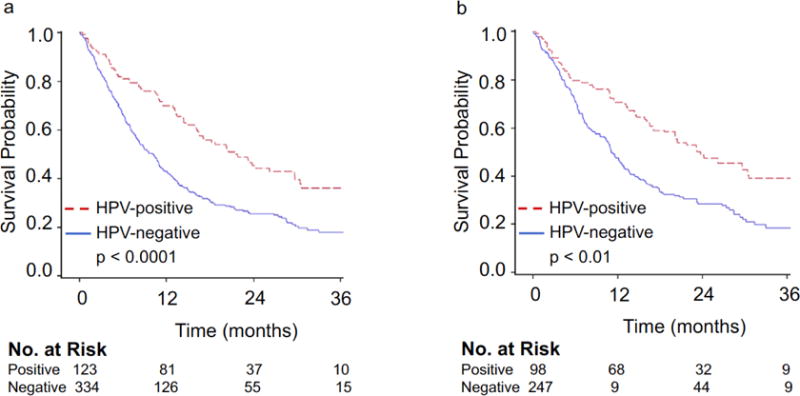
(a) Unadjusted and (b) adjusted Kaplan-Meier overall survival curves for all HPV-negative and HPV-positive Stage IVC non-OPSCC patients.

**Figure 2:**
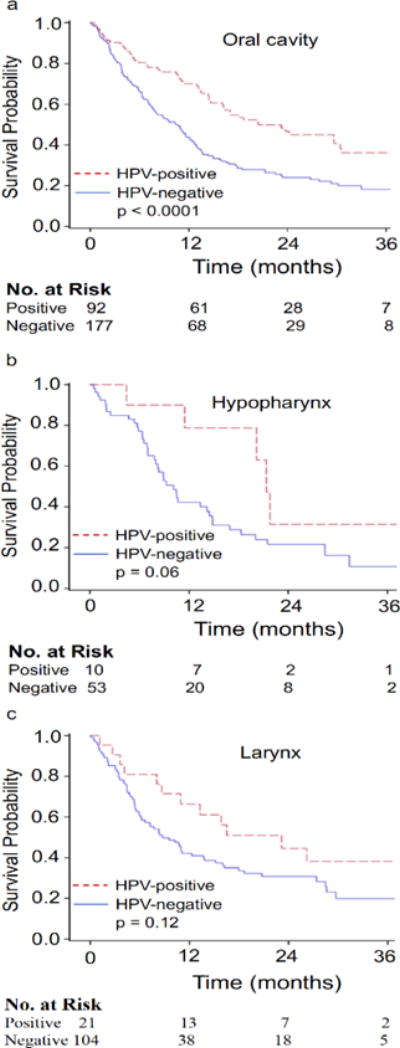
Unadjusted Kaplan-Meier overall survival curves for (a) oral cavity (b) hypopharynx, and (c) larynx HPV-negative and HPV-positive Stage IVC squamous cell carcinoma patients.

Unadjusted median survivals for HPV-negative and HPV-positive non-OPSCC patients were 10.3 months and 21.4 months, respectively, reaching statistical significance (p<0.0001) (Figure 1a). On multivariate analysis, receipt of any treatment and HPV-positive status were associated with a statistically significant improvement in overall survival whereas age ≥ 65 years and increasing tumor size were associated with statistically significant worse outcomes (Table 3). After adjusting for confounders, statistical significance for median survival was maintained at 11.1 months for patients with HPV-negative disease and 23.8 months for those testing positive for HPV (Figure 1b). Improved overall survival for patients with HPV-positive disease was maintained on sensitivity analysis (Table 4). On subsite analysis, unadjusted median survival for patients with HPV-negative or HPV-positive primary of the oral cavity was 10.4 months versus 20.5 months (p<0.0001), 10.1 months versus 21.4 months for hypopharynx (p=0.06), and 8.8 months versus 23.2 months for larynx (p=0.12), respectively (Figure 2a–2c). Propensity-score adjusted median survivals were similar to unadjusted median survivals with oral cavity at 11.6 months versus 20.5 months (p<0.05), 14.5 months versus 20.2 months for hypopharynx (p=0.23), and 10.3 months versus 41.2 months for larynx (p=0.09), respectively.

## Discussion

Differences in baseline patient characteristics between individuals with non-metastatic HPV-positive and HPV-negative OPSCC are well established and have recently been described for those with non-OPSCC [5,10,14,17]. Here, these differences were maintained in patients presenting with Stage IVC non-OPSCC. The most marked difference between the two patient cohorts was a median age at diagnosis of 64 years for HPV-negative and 59 years for HPV-positive patients, respectively. Also consistent with previously described non-metastatic HPV-positive patient characteristics, those with Stage IVC non-OPSCC HPV-positive cancer had fewer co-morbidities compared to the HPV-negative cohort. Unlike age which exhibited a significant association with survival outcomes, co-morbidity status was not associated with outcomes likely reflecting lack of granularity in the Charles/Deyo comorbidity index.

Patients with non-metastatic HPV-positive OPSCC or non-OPSCC commonly present with smaller primary tumors and harbor more extensive adenopathy compared to patients with HPV-negative disease [5,10,14,17]. Though there was a statistically significant distribution favoring higher T-stages in the HPV-negative cohort, the majority of patients, regardless of HPV-status, were diagnosed with ≥ T2 and ≥ N2b disease suggesting that both patient cohorts presented with advanced locoregional disease. These data suggest that distant metastatic disease typically occurs late in the natural history of the disease process regardless of HPV status.

Non-metastatic HPV-positive non-OPSCC patients exhibit improved outcomes compared to similar patients with HPV-negative disease [10-14]. Here, we demonstrate that patients with Stage IVC HPV-positive non-OPSCC exhibit a similar improvement in overall survival. Indeed, median survival for the HPV-negative cohort was approximately 10 months versus 21 months for those with HPV-positive disease. On multivariate analyses only, receipt of treatment and HPV-positive status was associated with an improved overall survival supporting the powerful prognostic implication of HPV status for patients with Stage IVC non-OPSCC.

The possible inclusion of patients with oropharyngeal primaries in the NCDB oral cavity patient cohort must be considered given the anatomic proximity of these head and neck regions. Whether or not the frequency of this potential reporting error occurs identically between the HPV-negative and HPV-positive subgroups is not able to be determined. The concern with this potential systematic error in data collection is the known favorable prognosis of the OPSCC patient cohort that could artificially improve the overall survival of the HPV-positive oral cavity cohort [1-5]. Therefore, caution should be applied in interpreting these data.

Limitations of the current study are those that are inherent to the data available in the NCDB. For example, routine HPV testing in non-OPSCC is not standard of care, as such, selection bias may exist in the data set. Therefore, factors driving the decision to test for HPV status may be contributing to the improved outcomes of the HPV-positive non-OPSCC cohort. Further, it needs to be noted that the NCDB used HPV-status and not the surrogate marker, p16. It remains to be determined if Stage IVC non-OPSCC HPV-positive patients exhibit similar outcomes as those that are p16-positive. Another limitation is the lack of outcomes data other than overall survival and any salvage therapies. It is known that p16-positive OPSCC exhibit improved outcomes following development of progressive disease [8]. Further investigation is therefore warranted in the HPV-positive non-OPSCC cohort to evaluate if similar trends may explain the current improvement of outcomes. Another significant limitation is the lack of tobacco use data. There is well established data demonstrating that smoking reduces the favorable prognosis associated with HPV status in OPSCC. Whether or not this holds true in the metastatic non-OPSCC remains to be defined ^5^.

## Conclusion

In this study, using a large national cancer database, we demonstrate improved overall survival for patients with HPV-positive non-OPSCC who present with metastatic disease at diagnosis. As the extent of metastatic disease at presentation, not captured by the NCDB, and variability in treatment demonstrated in this study may impact overall survival, caution needs to be applied to these findings. However, this hypothesis generating data demonstrating differences in overall survival for patients with Stage IVC HPV-positive versus HPV-negative non-OPSCC disease affords an opportunity for refinement of prognosis and consideration by head and neck oncologists as they provide cancer treatment recommendations to Stage IVC patients.

## Figures and Tables

**Table 1:** Patient demographics and disease characteristics for Patients with Stage IVC Non-OPSCC. RT: radiotherapy; CT: chemotherapy; CRT: chemoradiotherapy; NOS: not otherwise specified.

	HPV Negative	HPV Positive	All	p-value
Total patients	494	214	708	
**Age, years**				<0.001
Median (range)	64 (35-90)	59 (30-90)	62 (30-90)	
Mean (Std. Dev.)	63.9 (11.0)	60.3 (9.9)	62.8 (10.8)	
**Tumor Size (mm)**				0.78
Median (range)	38 (1-100)	38 (4-81)	38 (1-100)	
Mean (Std. Dev.)	39.3 (18.6)	38.2 (16.0)	39.0 (17.9)	
**Age**				<0.001
<65	260 (52.6)	147 (68.7)	407 (57.5)	
≥65	234 (47.4)	67 (31.3)	301 (42.5)	
**Sex**				<0.01
Male	384 (77.7)	186 (86.9)	570 (80.5)	
Female	110 (22.3)	28 (13.1)	138 (19.5)	
**Race**				0.12
White	370 (74.9)	180 (84.1)	550 (77.7)	
Black	80 (16.2)	20 (9.4)	100 (14.1)	
Hispanic	24 (4.9)	10 (4.7)	34 (4.8)	
Asian/Pacific islander	10 (2.0)	2 (0.9)	12 (1.7)	
Other	9 (1.8)	2 (0.9)	11 (1.6)	
Unknown	1 (0.2)	0 (0.0)	1 (0.1)	
**Year of Diagnosis**				0.08
2010	63 (12.8)	22 (10.3)	85 (12.0)	
2011	118 (23.9)	43 (20.1)	161 (22.7)	
2012	153 (31.0)	58 (27.1)	211 (29.8)	
2013	160 (32.4)	91 (42.5)	251 (35.5)	
**Charlson/Deyo Comorbidity Score**				0.05
0	336 (68.0)	165 (77.1)	501 (70.8)	
1	114 (23.1)	36 (16.8)	150 (21.2)	
≥ 2	44 (8.9)	13 (6.1)	57 (8.1)	
**Insurance Type**				<0.001
Private	118 (23.9)	95 (44.4)	213 (30.1)	
Medicare	225 (45.6)	74 (34.6)	299 (42.2)	
Medicaid	90 (18.2)	29 (13.6)	119 (16.8)	
Other Government	12 (2.4)	3 (1.4)	15 (2.1)	
No insurance	38 (7.7)	11 (5.1)	49 (6.9)	
Unknown	11 (2.2)	2 (0.9)	13 (1.8)	
**Income**				<0.05
<$48,000	233 (47.2)	79 (36.9)	312 (44.1)	
≥$48,000	256 (51.8)	133 (62.2)	389 (54.9)	
Unknown	5 (1.0)	2 (0.9)	7 (1.0)	
**Location**				0.78
Urban	379 (76.7)	163 (76.2)	542 (76.6)	
Non-urban	102 (20.7)	47 (22.0)	149 (21.1)	
Unknown	13 (2.6)	4 (1.9)	17 (2.4)	
**Facility**				0.62
Academic	244 (49.4)	110 (51.4)	354 (50.0)	
Community	250 (50.6)	104 (48.6)	354 (50.0)	
**Facility Volume**				0.12
High volume (Upper quartile)	99 (20.0)	54 (25.2)	153 (21.6)	
Low volume	395 (80.0)	160 (74.8)	555 (78.4)	
**Head and Neck Subsite**				<0.001
Oral Cavity	254 (51.4)	161 (75.2)	415 (58.6)	
Hypopharynx	78 (15.8)	20 (9.4)	98 (13.8)	
Larynx	162 (32.8)	33 (15.4)	195 (27.5)	
**Dose**				<0.01
None	231 (46.8)	81 (37.9)	312 (44.1)	
Low (50-65Gy)	32 (6.5)	24 (11.2)	56 (7.9)	
High (65-80Gy)	107 (21.7)	70 (32.7)	177 (25.0)	
Unknown	124 (25.1)	39 (18.2)	163 (23.0)	
**Primary Treatment**				<0.01
No treatment	83 (16.8)	21 (9.8)	104 (14.7)	
Surgery	27 (5.5)	2 (0.9)	29 (4.1)	
RT	50 (10.1)	14 (6.5)	64 (9.0)	
Surgery+RT	5 (1.0)	6 (2.8)	11 (1.6)	
CT	97 (19.6)	49 (22.9)	146 (20.6)	
CRT	179 (36.2)	96 (44.9)	275 (38.8)	
Surgery+CRT	25 (5.1)	12 (5.6)	37 (5.2)	
Unknown	28 (5.7)	14 (6.5)	42 (5.9)	
**T stage**				<0.05
T0	2 (0.4)	0 (0.0)	2 (0.3)	
T1	53 (10.7)	18 (8.4)	71 (10.0)	
T2	105 (21.3)	59 (27.6)	164 (23.2)	
T3	110 (22.3)	37 (17.3)	147 (20.8)	
T4	173 (35.0)	65 (30.4)	238 (33.6)	
Unknown	51 (10.3)	35 (16.4)	86 (12.2)	
**N stage**				0.15
	75 (15.2)	24 (11.2)	99 (14.0)	
N0	63 (12.8)	28 (13.1)	91 (12.9)	
N1	31 (6.3)	12 (5.6)	43 (6.1)	
N2 NOS	15 (3.0)	4 (1.9)	19 (2.7)	
N2A	103 (20.9)	52 (24.3)	155 (21.9)	
N2B	129 (26.1)	68 (31.8)	197 (27.8)	
N2C	46 (9.3)	20 (9.4)	66 (9.3)	
N3	32 (6.5)	6 (2.8)	38 (5.4)	
**HPV classification**				
HPV non-16-non-18 high-risk		13 (6.1)		
HPV-16 only		123 (57)		
HPV-18 only		2 (0.4)		
HPV-16 and HPV-18		11 (5.1)		
HPV high risk not stated		15 (7)		
HPV not otherwise specified		41 (19.2)		
HPV low risk		9 (4.2)		

**Table 2:** Multivariate logistics regression for probability of HPV-Positive disease among stage IVC non-OPSCC patients.

	OR	(95% CI)	p-value
**Age**			<0.01
<65	1.00	0.41-0.87	
≥65	0.60		
**Sex**			<0.01
Male	1.00	0.34-0.87	
Female	0.54		
**Insurance Type**			<0.01
Private	1.00	0.35-0.75	
Non-private/Unknown	0.52		
**Income**			<0.05
<$48,000	1.00	1.05-2.13	
≥ $48,000	1.50		
**Head and Neck Subsite**			<0.01 <0.001
Oral Cavity	1.00		
Hypopharynx	0.39	0.23-0.67	
Larynx	0.34	0.22-0.52	

**Table 3:** Univariate and multivariate analysis for overall survival of stage IVC Non-OPSCC patients using Cox proportional hazards model.

	Univariate			Multivariate		
	HR	(95% CI)	p-value	HR	(95% CI)	p-value
**Age**						<0.05
<65	1			1		
≥ 65	1.33	1.07-1.67	0.012	1.47	1.08-2.01	
**Sex**						
Male	1					
Female	0.92	0.70-1.21	0.538			
**Race**						
White	1					
Non-white	1.09	0.84-1.42	0.512			
**Year of Diagnosis**						
2010	1					
2011	1.21	0.89-1.65	0.226			
2012	1.14	0.84-1.55	0.407			
**Charlson/Deyo Comorbidity Score**						
0	1					
1	1.24	0.95-1.63	0.119			
≥ 2	1.61	1.09-2.39	0.017			
**Insurance Type**						
Private	1					
Non-private/Unknown	1.47	1.15-1.88	0.002			
**Income**						
<$48,000	1					
≥ $48,000	0.8	0.64-1.01	0.056			
**Location**						
Urban	1					
Non-urban	0.95	0.72-1.26	0.747			
**Facility**						
Community	1					
Academic	1.28	1.02-1.59	0.032			
**Volume**						
Low	1					
High	1.01	0.78-1.32	0.935			
**Head and Neck Subsite**						
Oral Cavity	1					
Hypopharynx	1.08	0.78-1.50	0.647			
Larynx	1.09	0.84-1.41	0.506			
**Dose**						
None	1					
Low (50-65Gy)	0.41	0.24-0.69	0.001			
High (65-80Gy)	0.47	0.35-0.62	<0.001			
Tumor Size (cm)	1.12	1.03-1.22	0.012	1.13	1.03-1.25	<0.05
**Treatment**						
No treatment	1			1		
Treatment	0.29	0.22-0.39	<0.001	0.29	0.19-0.43	<0.001
**T stage**						
0/1	1					
2	0.96	0.63-1.46	0.843			
3	1.24	0.83-1.87	0.297			
4	1.44	0.99-2.09	0.055			
**N stage**						
0	1					
1	1.19	0.76-1.87	0.437			
2	1.45	1.01-2.08	0.043			
3	1.42	0.86-2.34	0.166			
**HPV-status**						
Negative	1			1		
Positive	0.54	0.42-0.71	<0.001	0.43	0.29-0.64	<0.001

**Table 4:** Sensitivity analysis for overall survival of stage IVC Non-OPSCC patients using multivariate Cox proportional hazards model and multiple imputation for unknown HPV status.

	HR	(95% CI)	p-value
**Age**			<0.05
<65	1.00	1.04-1.46	
≥ 65	1.23		
Tumor Size (cm)	1.11	1.06-1.17	<0.001
(continuous)			
**Treatment**			<0.001
No treatment	1.00	0.16-0.24	
Treatment	0.20		
**HPV-status**		0.48-0.92	<0.01
Negative	1.00		
Positive	0.66		
